# Carga da doença, social e econômica do tabagismo no Brasil e impacto do aumento de impostos para a economia e para a redução da morbimortalidade

**DOI:** 10.1590/0102-311XPT002425

**Published:** 2026-06-26

**Authors:** Márcia Pinto, Ariel Bardach, Agustín Casarini, Federico Augustovski, Alfredo Palacios, Natalia Espinola, Marcia Gisele Santos, Kátia Marie Simões e Senna, Ana Clara de Moraes, Vera Luiza da Costa e Silva, Felipe Mendes, Cristiane Galhardo Ferreira Vianna, Ana Paula Leal Teixeira, Andres Pichon-Riviere

**Affiliations:** 1 Instituto Nacional de Saúde da Mulher, da Criança e do Adolescente Fernandes Figueira, Fundação Oswaldo Cruz, Rio de Janeiro, Brasil.; 2 Instituto de Efectividad Clínica y Sanitaria, Buenos Aires, Argentina.; 3 Centre for Health Economics, University of York, York, U.K.; 4 Instituto Nacional de Cardiologia, Rio de Janeiro, Brasil.; 5 Universidade Federal de Juiz de Fora, Juiz de Fora, Brasil.; 6 Secretaria-Executiva da Comissão Nacional para Implementação da Convenção-Quadro para o Controle do Tabaco, Instituto Nacional de Câncer, Rio de Janeiro, Brasil.

**Keywords:** Tabagismo, Avaliação Econômica, Cuidadores, Produtividade, Impostos, Tobacco Use Disorder, Economic Evaluation, Caregivers, Productivity, Taxes, Tabaquismo, Evaluación Económica, Cuidadores, Productividad, Impuestos

## Abstract

Este estudo buscou estimar a carga da doença, social e econômica do tabagismo no Brasil em 2022 e prever os benefícios do aumento de impostos, com a inclusão dos efeitos do mercado ilegal. Aplicou-se um modelo de microsimulação probabilística de Markov no qual indivíduos a partir de 35 anos foram acompanhados em coortes hipotéticas, quantificando as mortes, os eventos de saúde, o custo direto da assistência médica e o custo indireto por perda de produtividade. Foi desenvolvido um modelo para estimar os ganhos em termos de saúde e econômicos a partir de cenários de aumento de impostos em dez anos. Obteve-se os dados de prevalência do tabagismo, da estrutura demográfica brasileira, de mortalidade e morbidade em bases de dados oficiais e na literatura. Os recursos de saúde utilizados foram obtidos por meio da literatura e de consultas a especialistas. O tabagismo causou 174 mil mortes e 5,7 milhões de anos de vida perdidos por morte prematura e por incapacidade. A carga econômica foi de R$ 153 bilhões, sendo R$ 67 bilhões atribuídos ao custo direto e R$ 45 bilhões ao custo indireto. O custo do cuidador informal alcançou R$ 41 bilhões. No cenário com o mercado ilegal de produtos do tabaco, um aumento de 50% no preço dos cigarros tem o potencial de gerar benefícios econômicos de R$ 161 bilhões em dez anos. A Reforma Tributária atualmente em curso é oportuna para que o país possua uma política de preços e impostos permanente.

## Abstract (en)

This study aimed to estimate the disease, social, and economic burden of smoking in Brazil in 2022 and to predict the benefits of tax increases, including its effects of the illegal market. A probabilistic Markov microsimulation model was applied in which individuals aged 35 years or older were followed in hypothetical cohorts and quantified deaths, health events, the direct cost of medical care, and the indirect cost due to productivity losses. A model to estimate health and economic gains from 10-year tax increase scenarios was developed. Data on smoking prevalence, the Brazilian demographic structure, mortality, and morbidity were obtained from official databases and the literature. The used health resources were obtained from the literature and consultations with specialists. Smoking caused 174,000 deaths and 5.7 million years of life lost due to premature death and disability. Its economic burden totaled BRL 153 billion, of which BRL 67 billion was attributed to direct costs and BRL 45 billion to indirect costs. The cost of informal caregivers equaled BRL 41 billion. In the scenario with the illegal market for tobacco products, a 50% increase in the price of cigarettes may generate economic benefits of BRL 161 billion in 10 years. The current tax reform currently in Brazil may constitute a permanent pricing and tax policy.

## Abstract (es)

El objetivo de este estudio fue estimar la carga de la enfermedad, social y económica del tabaquismo en Brasil en 2022 y predecir los beneficios del aumento de los impuestos, incluidos los efectos del mercado ilegal. Se aplicó un modelo de microsimulación probabilística de Markov en el que se siguió a individuos de más de 35 años de edad en cohortes hipotéticas y se cuantificaron las muertes, los eventos de salud, el costo directo de la atención médica y el costo indirecto de la pérdida de productividad. Se desarrolló un modelo para estimar las ganancias sanitarias y económicas a partir de escenarios de aumento de impuestos a diez años. Los datos sobre la prevalencia del tabaquismo, la estructura demográfica brasileña, la mortalidad y la morbilidad se obtuvieron de bases de datos oficiales y de la literatura. Los recursos sanitarios utilizados se obtuvieron de la literatura y consultas con especialistas. El tabaquismo causó 174.000 muertes y 5,7 millones de años de vida perdidos por muerte prematura y discapacidad. La carga económica fue de BRL 153 mil millones, de los cuales BRL 67 mil millones se atribuyeron a costos directos y BRL 45 mil millones a costos indirectos. El costo del cuidador informal alcanzó los BRL 41 mil millones. En el escenario con el mercado ilegal de productos de tabaco, un aumento del 50% en el precio de los cigarrillos tiene el potencial de generar beneficios económicos de BRL 161 mil millones en diez años. La Reforma Tributaria actualmente en curso es oportuna para que el país tenga una política permanente de precios e impuestos.

## Introdução

O Brasil foi um dos países que mais reduziu a prevalência do tabagismo no período de 20 anos ^1^. Apesar desse resultado favorável e do avanço das medidas de controle do tabagismo, a carga da doença ainda é elevada - com cerca de 156 mil mortes anuais na última década − e concentrada nas doenças cardíacas, cerebrovasculares, doenças pulmonares obstrutivas crônicas (DPOC) e no câncer de pulmão ^2^.

A carga econômica global alcança anualmente USD 1,4 trilhão (R$ 8,4 trilhões) ou 1,8% do produto interno bruto (PIB) ^3^. No Brasil, o tabagismo é responsável por perdas anuais de USD 11,8 bilhões (R$ 72 bilhões), relacionadas à assistência médica, ao custo indireto por perda de produtividade, baixa produtividade e pela saída precoce do mercado de trabalho devido à morbidade ou à mortalidade ^4^. Agrega-se a esta carga o custo do cuidador informal de pacientes acometidos por doenças tabaco-relacionadas, que frequentemente necessita reduzir o tempo de trabalho, incorrendo em absenteísmo ou abandono das atividades laborais para se dedicar ao paciente que necessita de cuidados ^4^ ^,^ ^5^. No Brasil, estimativas apontam que a carga econômica do cuidador informal atribuível ao tabagismo alcançou USD 6 bilhões (R$ 30 bilhões) em 2020 ^6^.

O aumento de impostos é considerado uma medida altamente efetiva para a redução do consumo ^7^. No Brasil, teria o potencial de evitar quase 140 mil mortes e gerar um benefício econômico, expresso em custos evitáveis e aumento de arrecadação tributária de R$ 98 bilhões ao longo de 10 anos ^8^. No contexto brasileiro, às medidas de cunho fiscal, deve ser adicionada a análise do comportamento do mercado ilegal de cigarros e sua repercussão na saúde pública e na perda de arrecadação tributária ^9^.

Nesta pesquisa, em comparação com estudos prévios em que o mesmo método foi aplicado ^2^ ^,^ ^4^ ^,^ ^8^ ^,^ ^10^, foram incluídos o câncer de fígado, o diabetes tipo 2 e a tuberculose (TB). Estudos indicam que 18% dos casos de câncer de fígado podem ser atribuídos ao tabagismo ^11^. A doença cardíaca isquêmica, o acidente vascular cerebral (AVC) e a doença arterial periférica são as principais causas de mortalidade entre os pacientes com diabetes tipo 2 e o risco de morte na população em geral é quatro vezes maior quando o tabagismo está presente ^12^ ^,^ ^13^. Estima-se que 15,2% das mortes por TB em países de alta carga como o Brasil são atribuíveis ao tabagismo ^14^.

Este estudo estimou a carga do tabagismo no Brasil, sob a perspectiva da sociedade, em termos de mortalidade, morbidade, custos diretos da assistência médica, custos indiretos associados à perda de produtividade laboral e custos dos cuidadores informais. Estimou-se, ainda, como um aumento de impostos ao longo de um período de dez anos poderia reduzir o número de óbitos e de eventos associados às doenças tabaco-relacionadas, bem como aumentar a arrecadação de impostos em cenários com e sem a inclusão do comércio ilícito de cigarros observado no Brasil.

## Materiais e métodos

### Modelo de carga da doença associada ao tabagismo

Trata-se de um modelo econômico de estados de transição, ou modelo de Markov com microsimulação de Monte Carlo de primeira ordem, que, por meio de uma simulação probabilística individual, incorporou a história natural, os custos diretos e indiretos por perda de produtividade e perda de qualidade de vida associadas às doenças tabaco-relacionadas. O modelo foi desenvolvido no software Microsoft Excel (https://products.office.com/) ^2^ ^,^ ^15^ e seu desenvolvimento metodológico está apresentado em estudos previamente publicados ^2^ ^,^ ^4^ ^,^ ^8^ ^,^ ^10^.

As doenças selecionadas foram: AVC, diabetes tipo 2, doenças cardíacas isquêmicas e não isquêmicas (para este grupo, ver Material Suplementar; https://cadernos.ensp.fiocruz.br/static//arquivo/suppl-e002425_7341.pdf), doenças cerebrovasculares, DPOC, pneumonia e influenza, TB e os seguintes tipos de câncer: bexiga, boca e faringe, colo do útero, esôfago, estômago, fígado, laringe, pâncreas, pulmão, rins e pelve renal e leucemia mieloide. A pneumonia, a influenza e a TB possuem etiologia de origem infecciosa e sua inclusão justifica-se pelo aumento do risco entre fumantes de episódios graves de exacerbação. A estimativa da carga dessas três condições foi apresentada de forma agrupada nos resultados.

No modelo, os indivíduos foram seguidos dos 35 anos até a morte, em coortes hipotéticas, nas quais se estimou, em ciclos anuais, o risco de ocorrência de cada evento (episódio de DPOC, caso de câncer, de AVC etc.), conforme as características demográficas, o status do tabagismo (não fumante, fumante ou ex-fumante), condições clínicas e equações de risco, descritas em Pichon-Riviere et al. ^15^. Para cada indivíduo, por idade e sexo, foi calculado o risco basal de ocorrência a cada ano de eventos agudos e crônicos que poderiam estar relacionados à doença, sua progressão ou óbito, e os custos diretos (assistência à saúde) e indiretos (produtividade perdida por morte prematura e incapacidade).

Há escassez de dados de qualidade sobre a incidência populacional das doenças incluídas no modelo. Aplicou-se um método apoiado nos dados de mortalidade de 2022 do Sistema de Informações sobre Mortalidade (SIM; http://www.datasus.gov.br), adotado em modelos econômicos e epidemiológicos, que permite estimar os riscos absolutos por idade e sexo para eventos agudos ou crônicos ^16^ ^,^ ^17^ ^,^ ^18^ ^,^ ^19^ ^,^ ^20^. Há evidências de que as estatísticas nacionais podem subestimar a mortalidade por DPOC ^21^ ^,^ ^22^ e, por isto, as estimativas de risco, incidência e progressão da doença foram obtidas na literatura internacional ^23^ ^,^ ^24^. Estimou-se a letalidade por meio das hospitalizações registradas em 2022 no Sistema de Informações Hospitalares do Sistema Único de Saúde (SIH/SUS; http://www.datasus.gov.br).

O modelo registrou a frequência de desfechos mediante a probabilidade de cada indivíduo apresentar nenhum, um ou múltiplos eventos, pois os eventos agudos e as condições crônicas não são mutuamente excludentes. Adicionalmente, calculou-se a métrica dos anos de vida perdidos (AVP), composta pelos AVP por morte prematura (AVP-MP) e AVP por incapacidade (AVPI), os custos diretos e indiretos associados às doenças tabaco-relacionadas ^4^. O modelo não incluiu uma estimativa direta dos efeitos do tabagismo passivo e das causas perinatais (baixo peso ao nascer ou baixo tamanho ao nascer, síndrome da angústia respiratória e síndrome da morte súbita em recém-nascido). Utilizou-se uma aproximação baseada na literatura para o cálculo da mortalidade e a carga adicional para essas causas foi de 13,6% em homens e 12% em mulheres ^25^.

Realizou-se o processo de calibração e validação do modelo pela comparação da mortalidade específica por doença prevista pelo modelo, por sexo e idade, com as estatísticas nacionais, e, em seguida, procedeu-se com a análise dos desvios. As taxas médias de eventos simulados que se encontravam entre ±10% da taxa média dos eventos de referência (estatísticas e bases nacionais) foram consideradas aceitáveis e, para desvios maiores, as equações de risco ^15^ foram ajustadas para proporcionar um melhor ajuste dos resultados. Os resultados foram validados externamente com base em estudos epidemiológicos e clínicos não utilizados na estimativa e desenvolvimento das equações ^15^.

### Obtenção dos dados

Os parâmetros adotados no modelo têm como referência o risco individual de morte por cada causa ^15^, a estrutura demográfica brasileira e a prevalência de tabagismo conforme a *Pesquisa Nacional de Saúde* (PNS) de 2019 (https://www.ibge.gov.br/estatisticas/sociais/saude/9160-pesquisa-nacional-de-saude.html). Obteve-se os dados demográficos por meio da projeção da população para 2022 do Instituto Brasileiro de Geografia e Estatística (IBGE) (https://www.ibge.gov.br/estatisticas/sociais/populacao/9109-projecao-da-populacao.html?=&t=resultados), considerando-se cada indivíduo das coortes, por sexo e idade, a partir dos 35 anos. A mortalidade geral da população e a mortalidade específica por doença, idade e sexo foram incorporadas na estimativa do risco de morte, a partir dos dados SIM, que inclui informações dos indivíduos que acessam o Sistema Único de Saúde (SUS) e/ou a saúde suplementar. Os dados foram ajustados por meio de: (i) correção do sub-registro de óbitos pela aplicação de fator de ajuste da cobertura de óbitos ^26^; (ii) imputação dos valores ignorados para idade e sexo, substituídos respectivamente pela idade mediana e pelo sexo mais frequente calculados considerando-se os registros com a mesma causa básica; e (iii) redistribuição dos códigos-lixo e das causas mal definidas que não permitiam a classificação precisa da causa de óbito para as doenças incluídas no modelo (Projeto Carga de Doença, Escola Nacional de Saúde Pública Sergio Arouca, Fundação Oswaldo Cruz; http://www4.ensp.fiocruz.br/projetos/carga/downloads1.htm).

Obteve-se o risco relativo de desenvolver cada doença em fumantes e ex-fumantes em relação aos não fumantes no estudo de coorte *Cancer Prevention Study II* (CPS-II; II Estudo de Prevenção do Câncer) ^27^. A letalidade calculada pelo modelo para determinadas condições como infarto agudo do miocárdio (IAM), angina pectoris e AVC foi comparada com as estatísticas nacionais disponíveis de doença isquêmica coronariana ou de doenças cerebrovasculares. O risco absoluto de mortalidade por causa, sexo e idade foi obtido ao se dividir os dados de óbitos agrupados pelos códigos da 10ª revisão da Classificação Internacional de Doenças (CID-10) pela população brasileira acima dos 35 anos. Estimou-se a letalidade das neoplasias pelos prognósticos específicos por tipo de câncer, idade e sexo do Globocan ^20^ ^,^ ^28^. Os dados de hospitalizações por sexo, idade e código CID-10 da saúde suplementar não estavam disponibilizados publicamente e, por isso, realizou-se uma correção da base de dados do SIH/SUS a fim de incluir todas as hospitalizações ocorridas em 2022 no país ^29^.

### Estimativa da carga da doença associada ao tabagismo

A carga foi estimada pelas diferenças de ocorrência de eventos agudos e crônicos, mortes e custos entre os resultados preditos pelo modelo, conforme os dados atuais de prevalência do tabagismo da PNS 2019 e os resultados preditos para uma coorte de não fumantes. Os AVP expressam a carga do tabagismo, que é a soma dos AVP-MP e AVPI ^4^ ^,^ ^15^. Calculou-se os AVP-MP por meio de uma metodologia padronizada e usou-se medidas de utilidade dos estados de saúde de cada doença para estimar os AVPI ^30^.

### Cálculo dos custos

Adotou-se a perspectiva da sociedade que incluiu o custo direto da assistência à saúde e o custo indireto associado à perda de produtividade por morte prematura e incapacidade. Os custos estão apresentados em Reais de 2022 e o valor do PIB foi obtido na base de dados da Organização para Cooperação e Desenvolvimento Econômico (http://www.oecd.org). Aplicou-se a taxa de desconto de 5% ao ano para os desfechos de saúde e para os custos ^31^.

### Custo direto

Estimou-se o custo direto médio unitário por doença para o SUS e para o setor de saúde suplementar por meio da técnica do microcusteio e do custo por procedimento. O custo total refere-se a toda assistência à saúde prestada aos indivíduos com as doenças tabaco-relacionadas, a partir dos 35 anos. Foram consultados especialistas das áreas de oncologia, cardiologia, pneumologia e neurologia para a identificação e quantificação dos recursos de saúde, bem como a literatura. Os itens de custo incluídos foram consultas, exames, hospitalização, procedimentos cirúrgicos e não cirúrgicos. Incorporou-se esse custo ao modelo que estimou o custo total da assistência ao simular a probabilidade de ocorrência dos eventos ao longo da vida de cada indivíduo.

Para a valoração dos recursos para o SUS foram consultados: (i) Sistema de Gerenciamento da Tabela de Procedimentos, Medicamentos e OPM (http://sigtap.datasus.gov.br); (ii) Banco de Preços em Saúde do Ministério da Saúde (http://aplicacao.saude.gov.br/bps/login.jsf); (iii) Painel de Compras do Governo Federal (http://paineldecompras.economia.gov.br); e (iv) Câmara de Regulação do Mercado de Medicamentos (http://portal.anvisa.gov.br/cmed). Foram revisados os relatórios de aprovação de incorporação de tecnologias para o SUS da Comissão Nacional de Incorporação de Tecnologias no Sistema Único de Saúde (CONITEC; http://conitec.gov.br) retroativos ao ano de 2016 e o Rol de Procedimentos da Agência Nacional de Saúde Suplementar (ANS; https://www.ans.gov.br) com as incorporações de tecnologias até o final de 2022. Estas tecnologias foram submetidas aos especialistas para que pudessem validar sua real utilização na rotina da assistência. Obteve-se o custo do câncer de laringe ^32^, câncer de esôfago ^32^, câncer de fígado ^33^ e TB ^34^ para o SUS na literatura. Para a valoração dos recursos de saúde referentes ao setor de saúde suplementar foi utilizado o Painel D-TISS da ANS (https://www.ans.gov.br), contemplando a competência referente a dezembro de 2021, período disponível à época da realização da pesquisa. O valor médio do Painel D-TISS foi assumido como o reembolso efetivamente realizado pelas operadoras de planos e seguros de saúde aos prestadores de serviços de saúde. Quando necessário, os custos foram atualizados até dezembro de 2022 pelo Índice de Preços ao Consumidor Amplo do Banco Central do Brasil (https://www.bcb.gov.br/).

### Custo indireto

Foram adotadas duas estimativas: (i) morte prematura de indivíduos; e (ii) redução da produtividade laboral, devido à ocorrência de um evento de saúde, quando o indivíduo se reincorpora ao trabalho após a doença (presenteísmo). A abordagem do capital humano foi aplicada para estimativa do custo indireto por perda de produtividade por morte prematura ^35^ ^,^ ^36^. Para estimar o custo indireto por morte prematura, utilizou-se o valor de uma vida estatística (VSL, do inglês *value of a statistical life*) ^37^. A formulação matemática do cálculo do custo indireto está disponibilizada no Material Suplementar (https://cadernos.ensp.fiocruz.br/static//arquivo/suppl-e002425_7341.pdf).

### Custo do cuidador informal

O cuidador informal foi definido como o indivíduo responsável por prover cuidados sem receber nenhuma remuneração ou compensação financeira por este trabalho ^38^. Até onde conhecemos, não há estatísticas nacionais que informem o tempo gasto pelo cuidador informal na rotina diária de pessoas adoecidas. Realizou-se uma revisão da literatura para identificar as horas diárias dedicadas pelo cuidador aos pacientes com doenças tabaco-relacionadas. Obteve-se uma estimativa dessas horas que foram validadas com cuidadores formais e, em seguida, com cardiologistas, oncologistas e pneumologistas. Em uma terceira etapa, foram ajustados os valores das horas dedicadas diariamente pela aplicação de uma taxa de utilização do cuidado e do número de dias de cuidado por ano, conforme a gravidade da doença e se a condição era crônica ou aguda ^6^ (Material Suplementar; https://cadernos.ensp.fiocruz.br/static//arquivo/suppl-e002425_7341.pdf). Aplicou-se o *proxy good method* para estimar o valor do uso do tempo de um cuidador informal, no qual a média do salário-hora dos profissionais que atuam como assistentes sociais na área da saúde foi assumida como *proxy* do preço-sombra da hora de trabalho dos cuidadores informais no Brasil. O salário-hora foi estimado em R$ 21,00. Obteve-se a carga econômica para cada doença, a partir da multiplicação do salário-hora anual pelo total de casos. A revisão da literatura e o desenvolvimento do método estão detalhados em Espinola et al. ^6^.

### Modelo de impostos sobre cigarros e redução da mortalidade e morbidade

Foi elaborado com base na aplicação de percentuais de aumentos sobre os preços dos cigarros a partir de dois cenários que consideraram o mercado ilegal no Brasil. A elasticidade-preço da demanda foi de -0,48 (intervalo de -0,60 a -0,40) e o percentual do preço que seria atribuído aos impostos foi de 83% ^39^ ^,^ ^40^. O cálculo para estimar a prevalência de tabagismo após esse aumento encontra-se no Material Suplementar (https://cadernos.ensp.fiocruz.br/static//arquivo/suppl-e002425_7341.pdf).

Foram estabelecidos três cenários de aumento de preços de 25%, 50% e 75% ao longo de dez anos. Esses aumentos ocorreriam no curto, médio e longo prazo e permitiram medir o impacto acumulado nos desfechos de saúde. O caso de referência para a comparação foi estimado unificando esses cenários e os resultados foram acumulados para dez anos. Assumiu-se uma evolução linear do cenário de curto prazo para o cenário de médio prazo no período de cinco anos e, em seguida, para o cenário no longo prazo entre os anos 6 e 10. A partir das estimativas de mudança na prevalência e na redistribuição que implicam na proporção de fumantes, ex-fumantes e não fumantes na população, estima-se novamente a carga de doenças tabaco-relacionadas esperada no país com base nessas novas condições, seguindo o mesmo método adotado para a estimativa basal da carga de doença. Calculou-se o impacto sobre os desfechos de saúde como a diferença observada entre ambas as estimativas de óbitos, ocorrência de eventos, perda de anos de vida e custos diretos e indiretos.

### Inclusão do mercado ilegal no modelo de impostos

Estimou-se o comportamento da prevalência de tabagismo devido a um potencial efeito de substituição do consumo de cigarros legais por ilegais após um aumento da taxação. A prevalência foi medida pela *proxy* da elasticidade-preço cruzada da demanda *ε*_*cp*_ , ponderada pelo tamanho do mercado ilegal no país (*α*).

Diante da escassez de dados na literatura nacional sobre a migração dos consumidores do mercado legal para o ilegal, foram consideradas duas estimativas com o objetivo de obter essa informação e realizar uma análise de sensibilidade dos parâmetros do modelo: (i) por meio do consumo de cigarros no mercado ilegal antes e após a reforma tributária do tabaco na Colômbia ^41^ para estimar a *proxy* de elasticidade-preço cruzada da demanda entre o mercado legal e ilegal, em que *ε*_*cp*_ foi de 0,17; e (ii) pela análise do efeito das mudanças no preço dos cigarros a partir da Proposta de Reforma Tributária e da alteração de preços no mercado legal e ilegal no Brasil. A elasticidade encontrada foi de -0,076 ^42^.

### Impacto na arrecadação tributária

A variação na arrecadação que se poderia esperar nos diferentes cenários de aumento de impostos dos cigarros foi estimada pela variação percentual na arrecadação e no consumo devido ao aumento do preço de venda. Considerou-se também o percentual do imposto que incidiria no preço inicial de venda ao consumidor ^43^.

### Impacto em desfechos de saúde e para a economia

Em dez anos, estimou-se o impacto de três cenários de aumento de preços dos cigarros (em 25%, 50% e 75% do preço final ao consumidor) nos desfechos de saúde pela queda na prevalência e, por consequência, nos óbitos e na quantidade de eventos evitados. O benefício econômico total foi calculado pelo AVP evitado e pela soma dos custos diretos e indiretos evitados com a arrecadação tributária incremental. Os valores estão apresentados em Reais de 2022.

### Considerações éticas

Para a realização deste estudo, foram acessadas bases de dados públicos. De acordo com a *Resolução nº 510/2016* do Conselho Nacional de Saúde, não foi necessário submeter o estudo à apreciação de comitê de ética em pesquisa.

## Resultados

A taxa média de eventos para cada parâmetro esteve dentro de 10% das taxas verificadas nas estatísticas nacionais, o que garantiu uma excelente validação interna. A avaliação da correlação entre os resultados observados e os esperados produziu valores de R^2^ entre 0,700 e 0,999 (ajuste perfeito = 1), o que indicou elevado grau de correlação. Na validação externa, houve uma correlação favorável entre os valores preditos do modelo e os observados nas referências selecionadas (as figuras com os resultados estão no Material Suplementar; https://cadernos.ensp.fiocruz.br/static//arquivo/suppl-e002425_7341.pdf).

### Mortes e eventos atribuíveis ao tabagismo

Ocorreram 173.935 óbitos atribuíveis ao tabagismo em 2022, que representaram 12% do total ocorrido no Brasil em pessoas com 35 anos ou mais. A maior carga de mortalidade foi atribuída ao sexo masculino (69%). Para ambos os sexos, as principais causas foram a DPOC (23%), o câncer de pulmão (15%) e o IAM e as doenças cardíacas isquêmicas (12%). O tabagismo passivo e outras causas perinatais totalizaram 12% das mortes. O número de eventos anuais atribuíveis ao tabagismo foi de 1,77 milhão, liderado por diabetes tipo 2 (55%) e por DPOC (26%) para ambos os sexos (Tabela 1).

**Tabela 1:** Carga de mortalidade de doenças atribuíveis ao tabagismo, por sexo. Brasil, 2022.

Doenças	Óbitos				Total	
	Mulheres (A)	%	Homens (B)	%	A+B	%
IAM e eventos isquêmicos do coração	5.672	10	16.056	13	21.728	12
Diabetes tipo 2	1.662	3	3.632	3	5.294	3
Eventos não-isquêmicos do coração	2.353	4	6.789	6	9.142	5
AVC	3.703	7	5.810	5	9.513	5
Câncer de pulmão	9.144	17	17.440	15	26.584	15
Pneumonias, influenza e TB	3.134	6	8.611	7	11.745	7
DPOC	17.234	31	23.333	20	40.567	23
Câncer de boca e faringe	718	1	4.885	4	5.603	3
Câncer de esôfago	1.097	2	5.814	5	6.911	4
Câncer de estômago	577	1	2.697	2	3.274	2
Câncer de pâncreas	1.219	2	1.368	1	2.587	1
Câncer de rins e bexiga	540	1	2.599	2	3.139	2
Câncer de laringe	456	1	4.007	3	4.462	3
Leucemia mieloide	181	*	691	1	872	1
Câncer de fígado	613	1	1.217	1	1.830	1
Câncer de colo de útero	673	1	-	-	673	*
Tabagismo passivo e outras causas	5877	11	14.133	12	20.010	12
Total	54.853	100	119.082	100	173.935	100

AVC: acidente vascular cerebral; DPOC: doença pulmonar obstrutiva crônica; IAM: infarto agudo do miocárdio; TB: tuberculose.

Fonte: elaboração própria.

* Valores inferiores a 1%.

### Anos de vida perdidos

A perda de anos de vida para ambos os sexos foi de 5,7 milhões ao ano, dos quais 75% por AVP-MP e com concentração na DPOC (23%), câncer de pulmão (17%), IAM e as doenças cardíacas isquêmicas (15%) e AVC (7%). Desse total, a maior perda de AVP-MP ocorreu entre os homens (74%), sendo que foi superior, proporcionalmente, entre as mulheres para DPOC (29%) e câncer de pulmão (17%) (Tabela 2).

**Tabela 2:** Anos de vida perdidos por morte prematura (AVP-MP) e por incapacidade (AVPI) atribuíveis ao tabagismo, por sexo. Brasil, 2022.

Doenças	AVP-MP *				Total	
	Mulheres (A)	%	Homens (B)	%	A+B	%
IAM e eventos isquêmicos do coração	166.518	11	490.400	16	659.862	15
Diabetes tipo 2	127.017	9	182.287	6	306.947	7
Eventos não-isquêmicos do coração	42.360	3	93.277	3	129.110	3
AVC	57.493	4	169.919	5	224.289	5
Câncer de pulmão	70.192	5	176.736	6	248.796	6
Pneumonias, influenza e TB	429.358	29	564.971	18	987.595	23
DPOC	244.683	17	423.967	13	733.101	17
Câncer de boca e faringe	19.823	1	158.929	5	176.847	4
Câncer de esôfago	29.085	2	172.278	5	192.423	5
Câncer de estômago	16.013	1	75.368	2	89.230	2
Câncer de pâncreas	30.843	2	40.336	1	71.150	2
Câncer de rins e bexiga	16.017	1	70.287	2	93.483	2
Câncer de laringe	11.890	1	114.202	4	133.779	3
Leucemia mieloide	5.366	**	18.575	1	28.824	1
Câncer de fígado	15.811	1	31.444	1	47.331	1
Câncer de colo de útero	25.444	2	-	-	25.738	1
Tabagismo passivo e outras causas	156.949	11	378.485	12	543.004	13
Total	1.464.862	100	3.161.460	100	4.262.322	100
Doenças	AVPI				Total	
	Mulheres (A)	%	Homens (B)	%	A+B	%
IAM e eventos isquêmicos do coração	39.413	7	120.546	14	159.959	11
Diabetes tipo 2	66.994	12	75.500	9	142.494	10
Eventos não-isquêmicos do coração	41.006	7	71.117	8	112.123	8
AVC	-	-	-	-	-	-
Câncer de pulmão	69	**	146	**	215	**
Pneumonias, influenza e TB	289.751	53	395.387	45	685.138	48
DPOC	29.016	5	39.533	4	68.549	5
Câncer de boca e faringe	3.330	1	21.473	2	24.803	2
Câncer de esôfago	3.582	1	15.845	2	19.427	1
Câncer de estômago	2.662	**	8.980	1	11.642	1
Câncer de pâncreas	4.347	1	4.989	1	9.336	1
Câncer de rins e bexiga	2.722	**	10.587	1	13.309	1
Câncer de laringe	1.612	**	10.931	1	12.543	1
Leucemia mieloide	441	**	1.368	**	1.808	**
Câncer de fígado	1.624	**	2.956	**	4.579	**
Câncer de colo de útero	4.022	1	-	-	4.022	**
Tabagismo passivo e outras causas	58.871	11	105.993	12	164.864	11
Total	549.462	100	885.351	100	1.434.813	100

AVC: acidente vascular cerebral; DPOC: doença pulmonar obstrutiva crônica; IAM: infarto agudo do miocárdio; TB: tuberculose.

Fonte: elaboração própria.

* Valores sem desconto;

** Valores inferiores a 1%.

### Custos diretos e indiretos

A carga econômica total atribuível ao tabagismo foi de R$ 153,29 bilhões e representou 1,55% do PIB de 2022. Desse total, R$ 67 bilhões (44%) foram atribuídos ao custo direto das doenças respiratórias (35%), AVC (23%), tabagismo passivo (11%) e doenças cardíacas (10%). Do total de R$ 44,8 bilhões do custo indireto, R$ 20,45 bilhões (46%) foram atribuídos à perda de produtividade devido à morte prematura e R$ 24,35 bilhões (54%) à perda de produtividade por incapacidade. O custo do cuidador informal representou um incremento na carga total de R$ 41,26 bilhões (26,9%) (Tabela 3).

**Tabela 3:** Carga econômica anual por sexo, doença e tipo de custo atribuível ao tabagismo. Brasil, 2022.

Tipo de custo */Sexo	IAM e eventos isquêmicos do coração (A)	AVC (B)	Doenças respiratórias ** (C)	Diabetes tipo 2 (D)	Câncer de pulmão (E)	Outros tipos de câncer *** (F)	Tabagismo passivo e outras causas (G)	Total (A+B+C+D+E+F+G)
Direto								
Mulher	1.633.201.780	7.437.396.571	9.959.855.386	1.313.889.164	1.633.509.302	985.097.986	2.755.554.023	24.085.302.432
Homem	4.880.481.638	8.332.039.588	13.616.674.376	2.275.201.789	3.113.892.639	4.326.017.940	4.970.025.884	36.633.852.216
Total	6.513.683.418	15.769.436.159	23.576.529.762	3.589.090.953	4.747.401.941	5.311.115.926	7.725.579.907	67.232.838.066
Produtividade								
Morte prematura								
Mulher	606.709.514	616.153.137	1.481.452.516	75.911.149	704.840.251	624.755.014	631.112.183	4.134.224.252
Homem	2.956.696.194	1.128.230.757	3.310.457.272	359.159.318	1.611.273.933	3.859.838.762	2.484.479.249	12.753.439.291
Total	3.563.405.708	1.744.383.894	4.791.909.788	435.070.467	2.316.114.185	4.484.593.776	3.115.591.432	20.451.069.250
Morbidade								
Mulher	483.706.418	1.203.299.131	3.356.712.285	717.476.656	602.892.803	591.648.094	1.426.336.340	8.382.071.727
Homem	2.319.759.393	1.841.653.730	8.315.016	1.740.837.804	1.009.591.695	6.920.157.638	2.123.077.706	15.963.392.982
Total	2.803.465.811	3.044.952.861	3.365.027.301	2.458.314.460	1.612.484.498	7.511.805.732	3.549.414.046	24.345.464.709
Cuidador informal								
Mulher	1.170.999.308	2.547.355.754	7.146.431.550	1.012.943.202	1.012.943.202	875.302.361	813.766.181	14.023.814.851
Homem	3.525.925.881	2.883.732.514	9.903.291.782	1.754.067.428	1.754.067.428	1.675.649.571	3.204.204.542	22.541.720.391
Total	4.696.925.189	5.431.088.268	17.049.723.332	2.767.010.630	2.550.951.932	4.017.970.723	4.748.790.357	41.262.460.431
Carga econômica total	-	-	-					153.291.832.456

AVC: acidente vascular cerebral; IAM: infarto agudo do miocárdio.

Fonte: elaboração própria.

* Custo total das doenças estimado pelo modelo e com aplicação de desconto de 5%;

** Inclui doença pulmonar obstrutiva crônica, influenza, pneumonia e tuberculose;

*** Inclui os seguintes tipos de câncer: bexiga, boca e faringe, colo do útero, esôfago, estômago, fígado, laringe, pâncreas, pulmão, rins e pelve renal e leucemia mieloide.

### Efeitos esperados do aumento de preços de venda de cigarros por meio do aumento de impostos

Em dez anos, no cenário de referência (que desconsidera o mercado ilegal), o impacto produzido pelo incremento no preço geraria benefícios econômicos entre R$ 90,9 bilhões (aumento de 25%) e R$ 246,8 bilhões (aumento de 75%). Um aumento de preço de 50% evitaria cerca de 145 mil mortes, o dobro para um aumento de 25% (72.538), e aumentaria a arrecadação tributária em R$ 26 bilhões. Um total de 5,5 milhões de AVP seriam evitados, bem como R$ 64 bilhões em custos diretos. Nos cenários com o mercado ilegal e aumento de 50%, para as estimativas 1 e 2, os benefícios econômicos se reduziriam para R$ 111,22 bilhões e R$ 161,35 bilhões, respectivamente. Este mesmo comportamento de queda pode ser observado nos desfechos de mortalidade, morbidade e custos (Tabela 4).

**Tabela 4:** Benefícios esperados em saúde e econômicos ao longo de dez anos com um aumento de 25%, 50% e 75% no preço dos cigarros por meio de impostos no Brasil.

Benefícios	Sem o mercado ilegal (referência)			Com mercado ilegal (estimativa 1 *)			Com mercado ilegal (estimativa 2 **)		
	25%	50%	75%	25%	50%	75%	25%	50%	75%
Mortes evitadas	72.538	145.077	217.615	41.989	83.978	125.968	52.531	105.062	157.594
Doenças cardíacas evitadas	56.709	113.417	170.126	32.826	65.652	98.478	35.739	71.478	107.217
AVC evitados	48.671	97.342	146.012	28.173	56.347	84.520	30.673	61.347	92.020
Novos casos de câncer evitados	33.177	66.355	99.532	19.205	38.410	57.614	20.909	41.818	62.727
Novos casos de DPOC evitados	135.364	270.729	406.093	78.356	156.712	235.069	85.309	170.619	255.928
AVP-MP e AVPI evitados	2.756.039	5.512.078	8.268.118	1.595.345	3.190.690	4.786.034	1.736.914	3.473.828	5.210.741
Custo direto da assistência médica evitado (R$ milhões)	32.001	64.002	96.003	18.524	37.048	55.572	24.743	49.486	74.229
Custo evitado por produtividade perdida de cuidadores informais (R$ milhões)	17.502	35.003	52.505	10.131	20.262	30.393	18.771	37.542	56.314
Perda evitada de produtividade (R$ milhões)	24.102	48.180	72.234	13.955	27.901	41.840	24.173	48.313	72.420
Aumento na arrecadação tributária (R$ milhões)	17.318	26.009	26.072	17.318	26.009	26.072	17.318	26.009	26.072
Benefício econômico total (R$ milhões)	90.923	173.195	246.814	59.928	111.220	153.876	85.006	161.350	229.034

AVC: acidente vascular cerebral; AVPI: anos de vida perdidos por incapacidade; AVP-MP: anos de vida perdidos por morte prematura; DPOC: doença pulmonar obstrutiva crônica.

Fonte: elaboração própria.

* Estimativa 1: considerou o consumo de cigarros no mercado ilegal antes e após a reforma tributária do tabaco na Colômbia ^41^ para estimar a *proxy* de elasticidade-preço cruzada da demanda por produtos derivados do tabaco entre o mercado legal e o ilegal;

** Estimativa 2: estimativa do efeito das mudanças no preço dos cigarros a partir da proposta de Reforma Tributária, e o efeito da alteração de preços no mercado legal e ilegal no Brasil ^42^.

## Discussão

O custo total estimado anual foi de R$ 153,29 bilhões, dos quais 44% referem-se aos custos diretos, um montante de magnitude significativa para o SUS e para a saúde suplementar.

Em 2022, ocorreram 173.936 óbitos atribuíveis ao tabagismo no Brasil. A carga de mortalidade concentrou-se no sexo masculino (69%) e tem se mantido elevada ao longo dos últimos anos, com aproximadamente mais de 150 mil óbitos anuais, como também é demonstrado em outras pesquisas ^44^. A DPOC, o câncer de pulmão, o IAM e os eventos cardíacos isquêmicos representaram 48% das mortes. Em estudos anteriores que aplicaram o mesmo modelo de carga, o AVC era uma das principais causas de morte e, em 2022, não se observou este mesmo comportamento (5%). A redução da taxa de mortalidade por AVC vem sendo observada no Brasil desde 2000 e, até 2018, na faixa etária entre 35 e 44 anos, houve uma queda de 52,1% em homens e de 53,2% em mulheres ^45^. Apesar desses resultados favoráveis, o AVC e as doenças cardiovasculares ainda são responsáveis por uma significativa carga econômica no Brasil ^46^.

O número de eventos anuais atribuíveis ao tabagismo foi liderado por diabetes tipo 2 (974.982). Na última década, houve um aumento da prevalência dessa condição em 24% ^47^. Este é um desafio para o Brasil, que experimenta duas cargas combinadas - tabagismo e diabetes tipo 2 − que demandam estratégias efetivas e eficientes para garantir o acesso à prevenção e à assistência médica, bem como a ampliação do financiamento em saúde.

O custo indireto em 2022 no Brasil devido à perda de produtividade por morte prematura e por redução de produtividade por presenteísmo superou R$ 44 bilhões. Em outros países, também se observa uma carga substancial, com valores bastante significativos ^48^. Neste estudo, houve um incremento na carga do tabagismo advinda do custo do cuidador informal de R$ 41,26 bilhões ao ano. Sugere-se que mais pesquisas sejam realizadas diante de suas repercussões que vão além do aspecto econômico, abrangendo questões de apoio mental, proteção financeira e social desses cuidadores.

A política de preços e impostos é uma medida custo-efetiva na redução da carga do tabagismo e na geração de benefício econômicos para a sociedade ^49^. Espera-se que a Reforma Tributária em curso, ao incluir a taxação de cigarros no grupo das denominadas sin taxes ou imposto seletivo, seja efetiva para a redução da carga econômica, social e de saúde que o tabagismo impõe ao país, bem como para o aumento da arrecadação tributária. A literatura também vem corroborando o potencial desse instrumento de política na América Latina na redução de danos à saúde com benefícios fiscais ^50^.

Nossos resultados mostraram que a arrecadação tributária, sem a inclusão do mercado ilegal, poderia aumentar em R$ 26 bilhões ao longo de dez anos em um cenário de aumento de 50% dos preços, considerando que o preço do maço de cigarros corresponderia a 83% de impostos. No escopo da Reforma Tributária, a incidência de maior taxação sobre o preço de venda reduziria anualmente em 30,8% o consumo de cigarros com o aumento de até R$ 5,4 bilhões na arrecadação ^43^. Com a migração de consumidores para o mercado ilegal, os benefícios potenciais se reduziriam ao longo do período ^43^. Nos cenários com o mercado ilegal, há benefícios potenciais no aumento de impostos ao longo de dez anos, porém inferiores a um cenário sem o mercado ilegal. Apesar de uma redução pontual nos últimos anos, o mercado ilegal de cigarros é uma realidade no Brasil, e atribui-se sua permanência à fragilidade no cumprimento da legislação, ao pouco comprometimento no controle da cadeia produtiva de cigarros, à própria cultura de compra de produtos ilegais e à ausência de cooperação com o Paraguai, país que é a principal fonte de cigarros ilícitos para o Brasil ^9^.

A aplicação do modelo tem se mostrado uniforme e reprodutível em mais de 12 países latinoamericanos ^2^ ^,^ ^4^ ^,^ ^8^ e o processo de calibração e validação neste estudo se mostrou robusto, o que garantiu a reprodutibilidade dos resultados. No entanto, algumas limitações devem ser consideradas. Foi necessário consultar a literatura internacional devido à indisponibilidade de dados de utilidade no Brasil para o cálculo da carga da doença expressa em AVPI. Devido à insuficiência de dados nacionais, na análise de custo do cuidador informal utilizou-se a média de horas dedicadas ao cuidado por doença disponível na literatura e complementada por uma consulta a especialistas argentinos. Apesar dessa limitação, o objetivo foi ampliar as evidências acerca desse custo para o Brasil. A correção das hospitalizações por um fator de ajuste para a saúde suplementar, desagregadas para sexo e idade, foi necessária devido à insuficiência de dados baseados na CID-10. Esta correção permitiu que se calculasse a letalidade, mortalidade e o custo direto no nível nacional. Outras estimativas de custo não foram incluídas, como pela perda de produtividade devido ao absenteísmo, uma vez que a literatura reporta maiores custos entre os fumantes quando comparados aos não-fumantes ^51^. Além disso, o custo para o meio-ambiente não foi estimado e sugere-se que seja incluído futuramente nos estudos de carga econômica. O Brasil é o primeiro exportador mundial de tabaco e as evidências mostram que a indústria do tabaco globalmente contribui sobremaneira para as mudanças climáticas com a elevada emissão anual de gases de efeito estufa ^52^.

## Conclusão

A carga do tabagismo é significativamente alta para o Brasil. O aumento de preços e impostos enquadra-se em uma possibilidade de aumento de arrecadação e redução de mortes, adoecimento e custo para a sociedade. Os resultados baseados nos cenários aqui apresentados fortalecem a sua custo-efetividade. Neste contexto, a Reforma Tributária tem papel crucial para o país avançar na redução da carga da doença e do impacto econômico e social do consumo de tabaco no Brasil. Ademais, a compensação pelos danos causados pelo tabagismo à sociedade deve ser efetivada para contribuir com a redução do custo de oportunidade que este fator de risco impõe à sociedade brasileira.

## Data Availability

Os dados de pesquisa estão disponíveis mediante solicitação à autora de correspondência.

## Material Suplementar

Material Suplementar
