# Supplementary material for: Disease, social, and economic burden of smoking in Brazil and the impact of tax increases on the economy and on the reduction of morbidity and mortality
Source: Cad Saude Publica. 2026 Jun 26;42:e00002425. [Article in Portuguese] doi: 10.1590/0102-311XPT002425 (PMC13313698; doi:10.1590/0102-311XPT002425)
Supplement: Material Suplementar [file 1678-4464-csp-42-PT002425-s.pdf]

Material Suplementar

S1. Condições relacionadas às doenças cardiovasculares não-isquêmicas

Essas doenças possuem ampla abrangência e referem-se às condições cardiovasculares que não são causadas por obstrução nos vasos sanguíneos que irrigam o coração. Inclui-se condições como doenças das válvulas cardíacas, cardiopatia reumática, miocardiopatias, cardiopatia hipertensiva, insuficiência cardíaca e outras. Os códigos da Classificação Internacional de Doenças utilizados no estudo estão listados a seguir.

I00; I010-I012; I018-I020; I029; I050-I052; I058; I062; I068-I072; I078-I083; I088-I092; I098-I099; I110; I119; I260; I269- I272; I278-I281; I288; I289; I300; I301; I308-I313; I318-I319; I320; I321; I328; I330; I339-I342; I348-I352; I358-I362; I368-I372; I378 ; I379; I38X; I390 -I394; I398; I400; I401; I408; I409; I410 -I412; I418; I420-I429; I43-I432; I438; I440-I447; I456; I458-I461; I469-I472; I479; I48X; I490-I495; I498-I501; I509-I519.

S2. Método de cálculo do custo indireto por perda de produtividade por morte prematura e por redução da capacidade laboral

Para estimar o custo por perda de produtividade devido à morte prematura, utilizou-se o valor de uma vida estatística (VSL, do inglês *value of a statistical life*). O valor presente da renda futura de uma pessoa depende da expectativa de vida, de sua participação no mercado de trabalho e da renda laboral. O valor do capital humano de um indivíduo de determinado sexo e idade é o valor presente dessa renda no futuro, dado pela fórmula atuarial do VSL[1]:

$$VSL = \sum_{j=i}^{E(x)} \text{prob}(\text{vivo}) * \text{Salário} * \left(\frac{1+g}{1+r}\right)^{E(x)-i} \qquad \text{Equação 1}$$

Onde: *prob(vivo)* é a probabilidade de que um indivíduo esteja vivo no próximo ano (IBGE. Projeção da população brasileira por sexo e idade: 2010-2060 https://www.ibge.gov.br/estatisticas/sociais/populacao); *salário* é uma estimativa da renda laboral anual do indivíduo obtida através da Pesquisa Nacional de Amostra por Domicílios Contínua, último trimestre de 2022. (https://www.ibge.gov.br/estatisticas/sociais/trabalho/17270-pnad-continua.html); e o último termo considera dois parâmetros assumidos como constantes: uma taxa de crescimento da renda laboral ao longo do tempo (parâmetro *g*), cuja premissa é a de que seria igual à taxa de crescimento médio anual do PIB *per capita* do Brasil de 2,09% ao ano, entre 1960 e 2022 (World Bank. http://data.worldbank.org/country/brazil) e um fator de desconto da

renda futura (parâmetro *r*) de 5%[2]. O cálculo do VSL associado a um indivíduo de determinado sexo e idade é a soma dos produtos destes termos para cada idade.

Foi necessário adotar métodos alternativos da estimativa da redução da produtividade laboral devido ao presenteísmo, utilizando-se um critério de estimativa indireta: assumiu-se que a produtividade laboral dos indivíduos diminui devido ao tabagismo na mesma proporção da redução da qualidade de vida que lhe é atribuída[3-6]. Adicionalmente, estimou-se a probabilidade do indivíduo sobreviver de um ano *t* a um ano *t+1* conforme a tábua de vida por idade e sexo de 2022 (http:www.ibge.gov.br).

Aplicou-se a equação de Mincer[7,8] para estimar a renda média anual (refere-se ao termo *salário* da Equação 1) por sexo e idade com base no nível máximo de escolaridade alcançado, da experiência no mercado de trabalho (aproximada pela idade e pela idade ao quadrado) e da localização geográfica:

$$\ln(\text{salário}) = \alpha + \beta\,\text{idade} + \gamma\,\text{idade}^2 + \delta\,\text{nível edu 1} + \ldots + \theta\,\text{nível edu 8} + \varphi\,\text{urbano} + \varepsilon$$

Equação 2

Onde: *ln (salário)* é o logaritmo natural do salário recebido pela atividade principal do indivíduo, *idade* e *idade*^{2} buscaram aproximar a experiência laboral, *nível edu 1*,... *nível edu 8* representam os distintos níveis de escolaridade alcançados, *urbano* é a variável que indica se vive em área urbana ou rural e *ε* representa o termo de erro do modelo, que se assume cumprir os pressupostos clássicos. Para o cálculo da equação, o método de mínimos quadrados ordinários foi aplicado e os dados obtidos por intermédio da PNAD Contínua do último trimestre de 2022 (https://www.ibge.gov.br/estatisticas/sociais/trabalho/17270-pnad-continua.html).

O modelo estimou o custo da renda futura do trabalho perdida de acordo com a idade de aposentadoria dos indivíduos, que foi fixada em 65 anos para homens e 62 anos para mulheres de acordo a Reforma da Previdência de 2019. As análises foram realizadas no *software* Stata.

Referências

1. Lev B, Schwartz. On the use of the economic concept of human capital in financial statements. The Accounting Review, 1971; 46(1), 103–112. http://www.jstor.org/stable/243891
2. Departamento de Tecnologia e Insumos Estratégicos, Secretaria de Ciência, Tecnologia e Insumos Estratégicos, Ministério da Saúde. Diretrizes metodológicas: estudos de avaliação econômica de tecnologias em saúde. Brasília: Ministério da Saúde; 2014.
3. Krol M, Brouwer W, Rutten F. Productivity costs in economic evaluations: past, present, future. PharmacoEconomics 2013;31(7):537-549.

4. Mattke S, Balakrishnan A, Bergamo G, Newberry SJ. A review of methods to measure health-related productivity loss. The American journal of managed care 2007; 13(4):211-217.
5. Knies S, Severens JL, Ament AJ, Evers SM. The transferability of valuing lost productivity across jurisdictions. differences between national pharmacoeconomic guidelines. Value in Health 2010;13(5):519-527.
6. Lamers LM, Meerding WJ, Severens JL, Brouwer WB. The relationship between productivity and health-related quality of life: an empirical exploration in persons with low back pain. Quality of life research 2005;14(3):805-813.
7. Lemieux T. The “Mincer equation” thirty years after schooling, experience and earnings. In: Grossbard S, editor. Jacob Mincer: a pioneer of modern labor economics. Boston: Springer; 2006. p. 127-45.
8. Harberger AC, Guillermo-Peón S. Estimating private returns to education in Mexico. Lat Am J Econ. 2011;49:1-35.

S3. Calibração e validação do modelo econômico

A taxa média de eventos para cada parâmetro incluído no modelo se manteve em 10% das taxas verificadas nas estatísticas nacionais, o que garantiu uma excelente validação interna. Houve um alto grau de correlação entre os resultados observados e os esperados ao produzir valores de R^2 entre 0,700 e 0,999 (ajuste perfeito = 1). A validação externa foi realizada pela comparação dos resultados do modelo com estudos epidemiológicos que não haviam sido utilizados como fonte de dados neste estudo. Novamente, houve uma correlação favorável entre os valores preditos do modelo e os observados nas referências selecionadas. Os resultados estão apresentados nas Figuras abaixo.

Figura 1. Calibração: Número anual de mortes predito pelo modelo comparado com as estatísticas nacionais e Globocan para quatro condições selecionadas: (a) Infarto agudo do miocárdio (mulheres); (b) câncer de rins (mulheres); (c) mortes por doenças cardiovasculares não-isquêmicas (homens) e; (d) câncer de pulmão (homens).

(a)

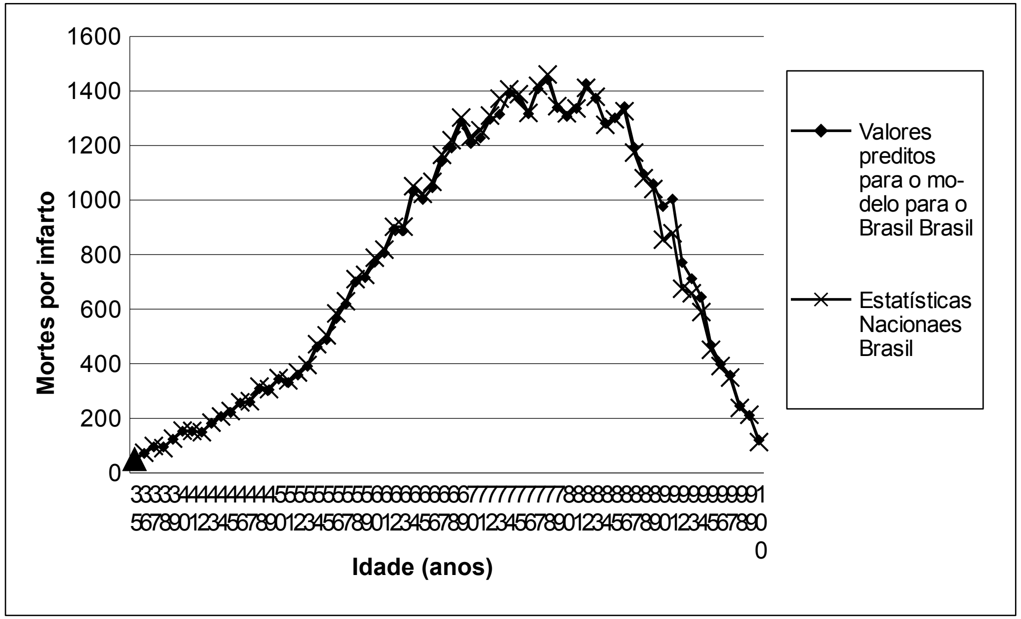

(b)

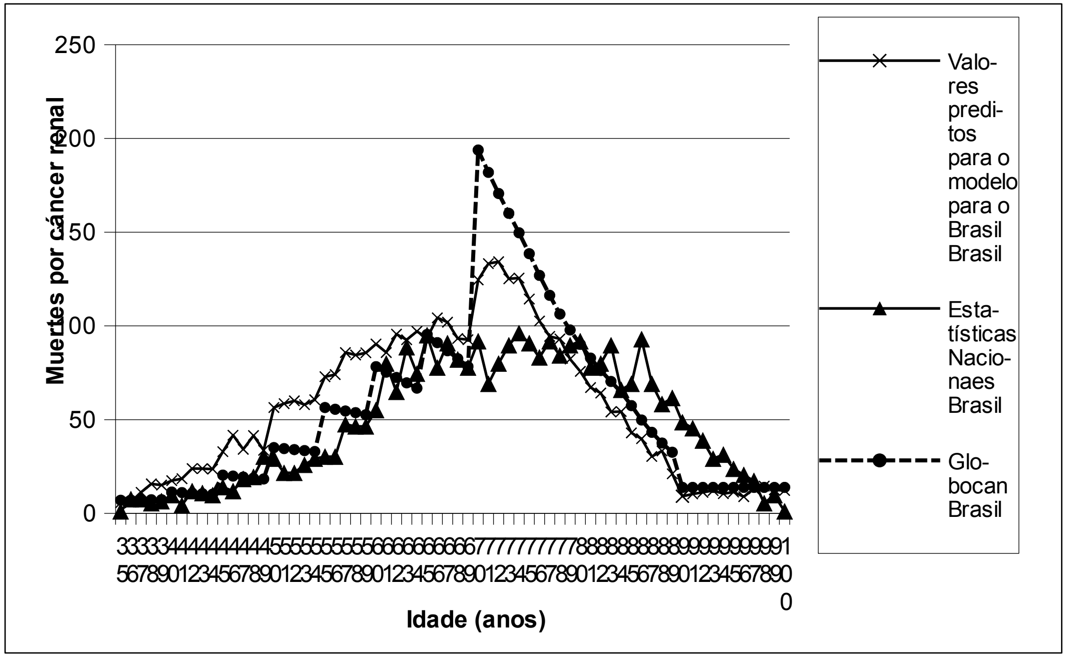

(c)

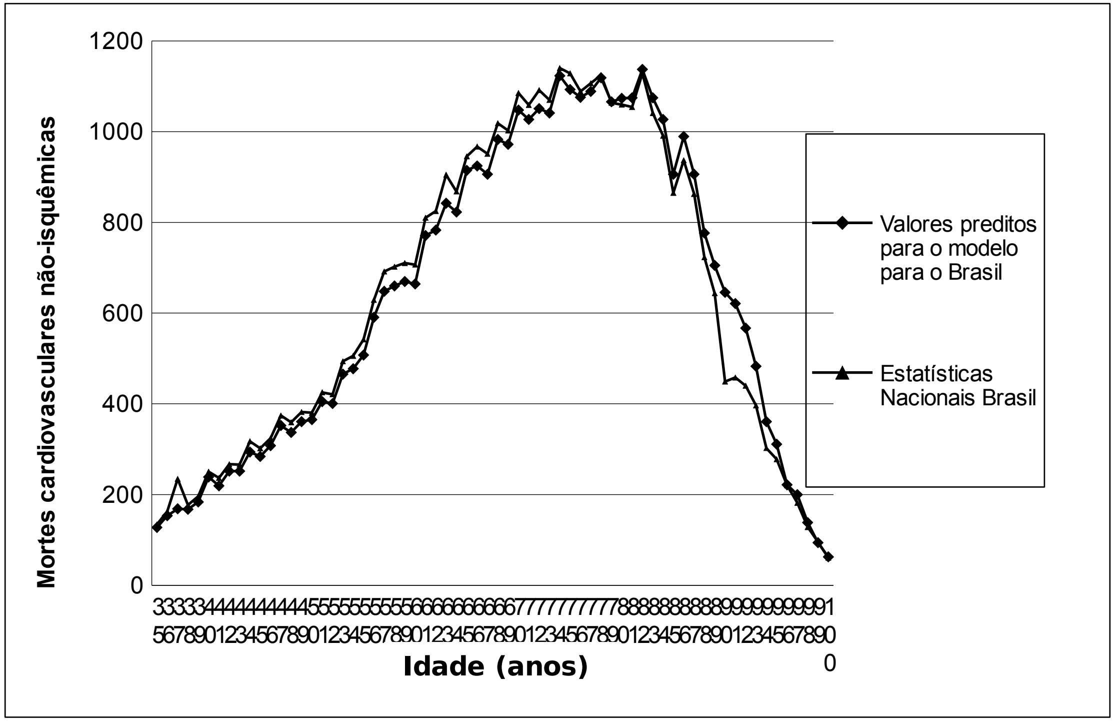
1200
1000
800
600
400
200
0
Mortes cardiovasculares não-isquêmicas
Idade (anos)
Valores preditos para o modelo para o Brasil
Estatísticas Nacionais Brasil

(d)

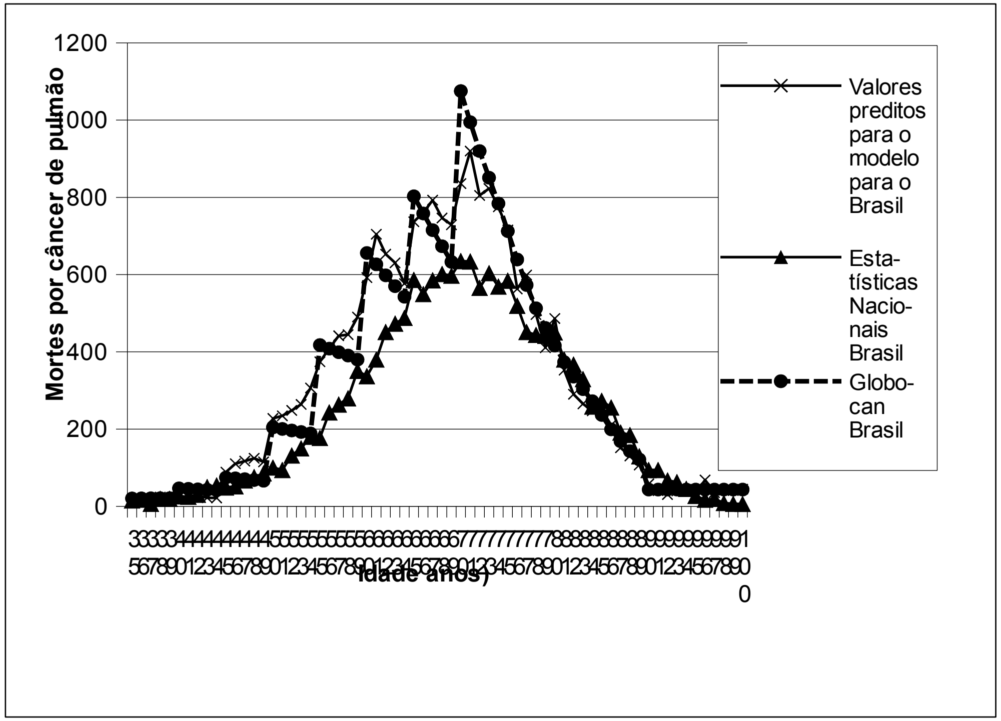
1200
1000
800
600
400
200
0
Mortes por câncer de pulmão
Idade anos)
Valores preditos para o modelo para o Brasil
Estatísticas Nacionais Brasil
Globocan Brasil

Figura 2. Gráfico de correlação de mortalidade entre os valores preditos pelo modelo aplicado no Brasil e os valores esperados de acordo com as estatísticas nacionais ou com o GLOBOCAN (*International Agency for Research on Cancer* - IARC) para quatro condições selecionadas:

(a) acidente vascular cerebral (AVC) em homens.

(b) câncer de pulmão em mulheres.

(c) câncer de esôfago em homens.

Os gradientes das linhas de regressão (y) e os coeficientes de correlação (R^2) estão apresentados em cada gráfico.

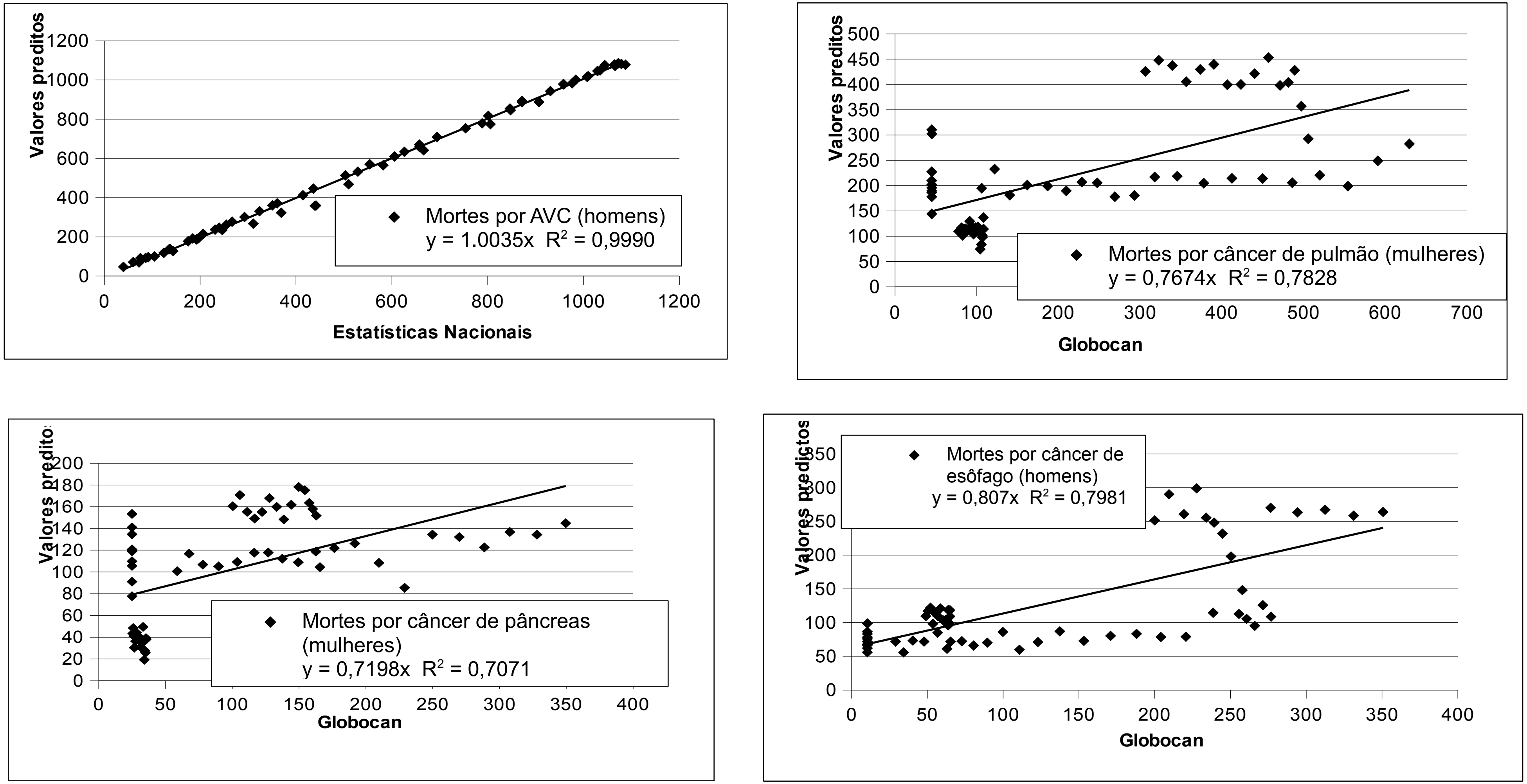

Figura 3. Validação com estudos epidemiológicos selecionados - Resultados correspondentes à população masculina:

(a) Incidência de infarto predita pelo modelo comparada com os estudos de incidência de base populacional: *Danish WHO MONICA study register* e estudo de incidência de infarto na Argentina (Coronel Suárez).

(b) Prevalência de DPOC predita pelo modelo comparada com a prevalência reportada pelo estudo PLATINO (*Latin American Project for the Investigation of Obstructive Lung Disease*).

(c) Incidência de acidente vascular cerebral predita pelo modelo comparada com o *WHO MONICA study register* em países selecionados (Finlândia - WHO *MONICA North Karelia province*, Rússia - *WHO MONICA Novosibirsk city*, e Lituânia - *WHO MONICA Kaunas city*).

(d) Incidência de câncer de pulmão predita pelo modelo comparada com as estimativas da IARC.

(e) Taxa de mortalidade por câncer de pulmão predita pelo modelo comparada com as estimativas realizadas pela IARC.

(a)

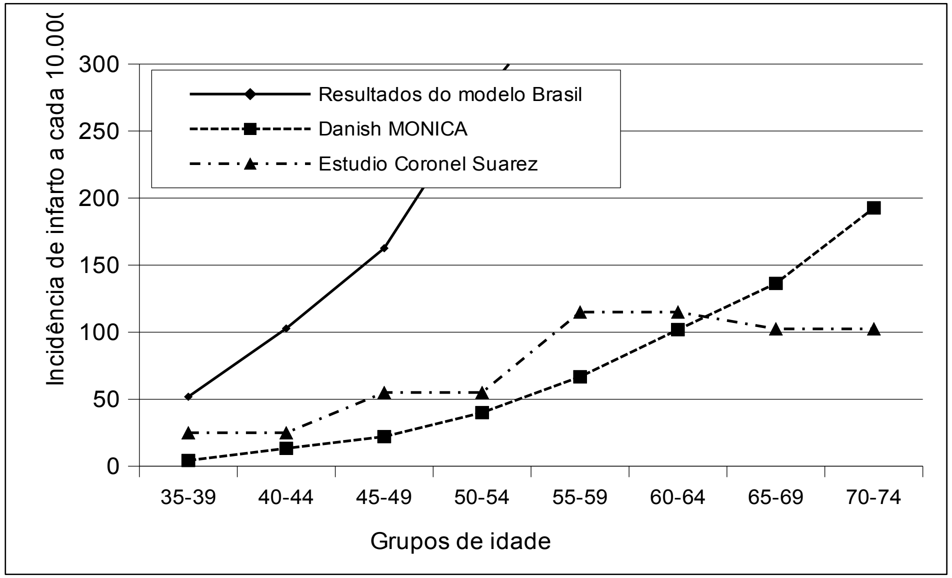

(b)

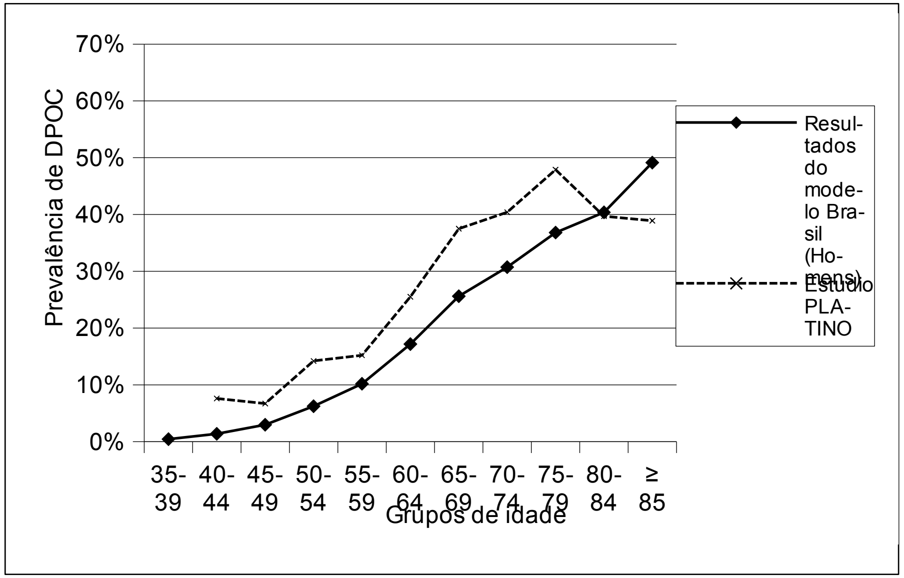

(c)

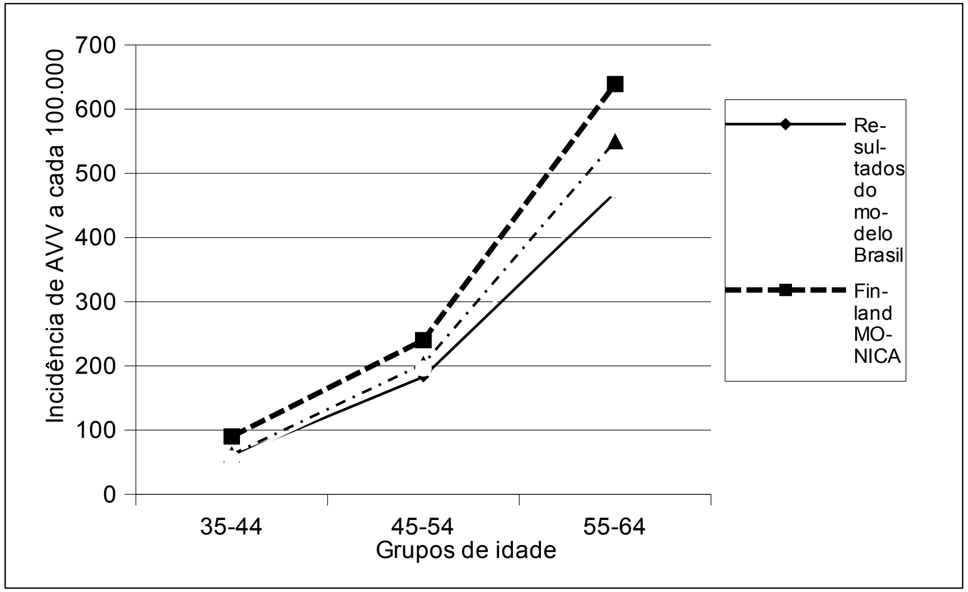

Incidência de AVV a cada 100.000
700
600
500
400
300
200
100
0
35-44
45-54
55-64
Grupos de idade
Re-sul-tados do mo-delo Brasil
Fin-land MO-NICA

(d)

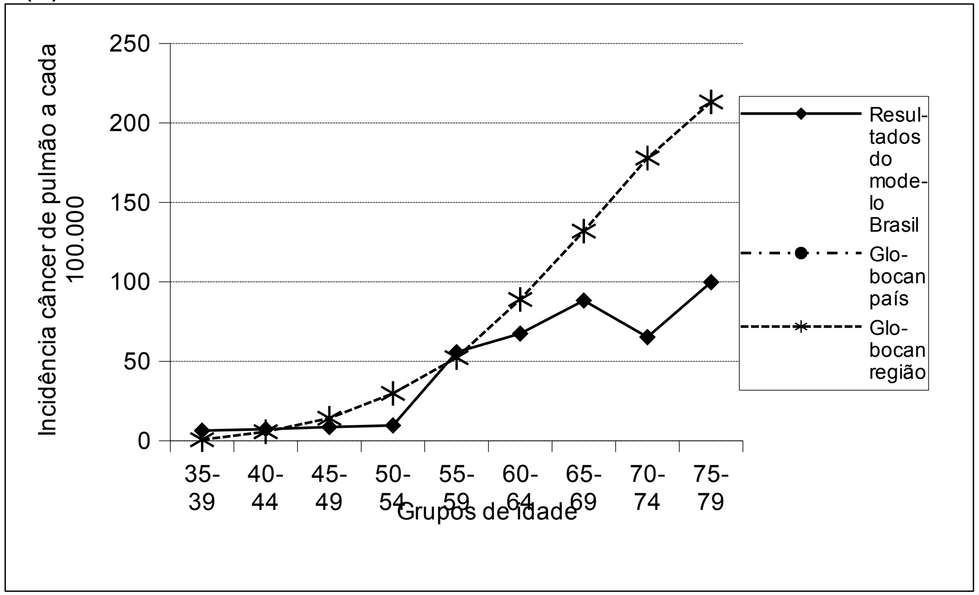

Incidência câncer de pulmão a cada 100.000
250
200
150
100
50
0
35-39
40-44
45-49
50-54
55-59
60-64
65-69
70-74
75-79
Grupos de idade
Resul-tados do mode-lo Brasil
Glo-bocan país
Glo-bocan região

(e)

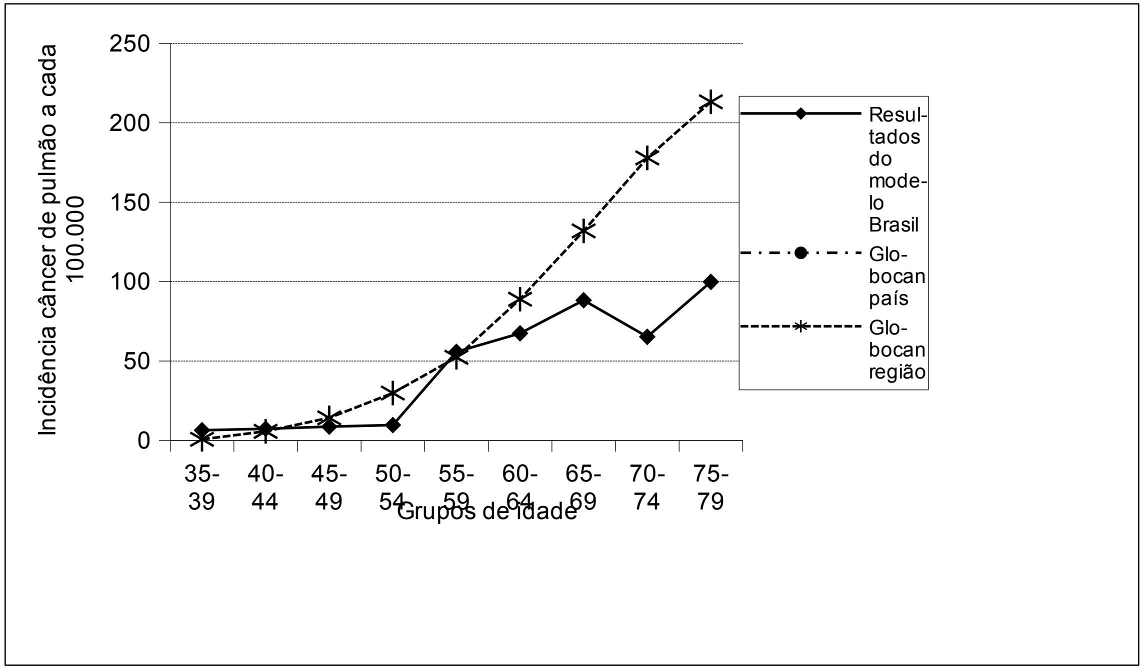

Incidência câncer de pulmão a cada 100.000
250
200
150
100
50
0
35-39
40-44
45-49
50-54
55-59
60-64
65-69
70-74
75-79
Grupos de idade
Resul-tados do mode-lo Brasil
Glo-bocan país
Glo-bocan região

S4. Modelo de impostos sobre cigarros e redução da mortalidade e morbidade

A fórmula matemática para estimar a prevalência de tabagismo obtida após o aumento do preço dos derivados do tabaco segue abaixo:

$$\text{Prevalência}_{post\text{-}tax} = \text{Prevalência}_{pre\text{-}tax} + (E_d * \Delta P\% * Ip * \text{Prevalência}_{pre\text{-}tax})$$

Em que: $\text{Prevalência}_{post\text{-}tax}$ = prevalência de tabagismo após o aumento de preços; $\text{Prevalência}_{pre\text{-}tax}$ é a prevalência de tabagismo antes do aumento de preços; E_d é a elasticidade-preço da demanda; Δ%P é a variação percentual do preço; e Ip é a proporção da variação no consumo que impacta na prevalência de fumantes.

S5. Parâmetros usados para estimar as horas diárias gastas pelos cuidadores informais por eventos.

Eventos	Utilidade	Referência	Dias de cuidado / ano[§]	Taxa de utilização do cuidado
IAM (1º ano)	0.803	Smith	30	65%
Evento coronariano, exceto IAM (1º ano)	0.803	Smith	30	65%
Evento coronariano (a partir do 2º ano)	0.850	Wijeysundera	365	5%
AVC (1º ano)	0.620	Yeoh	60	33%
AVC (a partir do 2º ano)	0.780	Yeoh	365	17%
DPOC leve	0.935	Mölken	365	0%
DPOC moderada	0.776	Mölken	365	60%
DPOC grave	0.689	Mölken	365	80%
Pneumonia	0.994	Pepper	30	65%
Câncer de pulmão	0.660	Chouaid	365	100%
Câncer oral	0.745	Nie	365	100%
Câncer de esôfago	0.630	Graham	365	100%
Câncer de estômago	0.550	Dan	365	100%
Câncer de pâncreas	0.550	Gordois	365	100%
Câncer de rins	0.780	Pickard	365	100%
Câncer de laringe	0.760	Pickard	365	100%
Leucemia	0.820	Leunis	365	100%
Câncer de bexiga	0.678	Hevér	365	100%
Continuação.				
Câncer de pescoço	0.758	Endarti	365	100%

IAM: infarto agudo do miocárdio; AVC: acidente vascular cerebral; DPOC: doença pulmonar obstrutiva crônica.

§ Equação resultante da estimativa econométrica para os dados faltantes (*missing data*) referentes às horas de cuidado informal: y= -13.32*x + 12.325

Referências

1. Smith DW, Davies EW, Wissinger E, Huelin R, Matza LS, Chung K. A systematic literature review of cardiovascular event utilities. Vol. 13. Expert Review of Pharmacoeconomics and Outcomes Research. 2013.

2. Wijeysundera HC, Farshchi-Zarabi S, Witteman W, Bennell MC. Conversion of the Seattle Angina questionnaire into EQ-5D utilities for ischemic heart disease: A systematic review and catalog of the literature. Clin Outcomes Res. 2014;6(1).

3. Yeoh YS, Koh GCH, Tan CS, Tu TM, Singh R, Chang HM, *et al.* Health-related quality of life loss associated with first-time stroke. PLoS One. 2019;14(1).

4. Mölken MR Van, Lee TA. Economic modeling in chronic obstructive pulmonary disease. In: Proceedings of the American Thoracic Society. 2006.

5. Pepper PV, Owens DK. Cost-effectiveness of the pneumococcal vaccine in healthy younger adults. Med Decis Mak. 2002;22(5 SUPPL.).

6. Chouaid C, Agulnik J, Goker E, Herder GJM, Lester JF, Vansteenkiste J, *et al.* Health-related quality of life and utility in patients with advanced non-small-cell lung cancer: A prospective cross-sectional patient survey in a real-world setting. J Thorac Oncol. 2013;8(8).

7. Nie M, Liu C, Pan YC, Jiang CX, Li BR, Yu XJ, *et al.* Development and evaluation of oral Cancer quality-of-life questionnaire (QOL-OC). BMC Cancer. 2018;18(1).

8. Graham AJ, Shrive FM, Ghali WA, Manns BJ, Grondin SC, Finley RJ, *et al.* Defining the Optimal Treatment of Locally Advanced Esophageal Cancer: A Systematic Review and Decision Analysis. Ann Thorac Surg. 2007;83(4).

9. Dan YY, So JBY, Yeoh KG. Endoscopic Screening for Gastric Cancer. Clin Gastroenterol Hepatol. 2006;4(6).

10. Gordois A, Scuffham P, Warren E, Ward S. Cost-utility analysis of imatinib mesilate for the treatment of advanced stage chronic myeloid leukaemia. Br J Cancer. 2003;89(4).

11. Pickard AS, Jiang R, Lin HW, Rosenbloom S, Cella D. Using Patient-reported Outcomes to Compare Relative Burden of Cancer: EQ-5D and Functional Assessment of Cancer Therapy-General in Eleven Types of Cancer. Clin Ther. 2016;38(4).

12. Leunis A, Redekop WK, Uyl-de Groot CA, Löwenberg B. Impaired health-related quality of life in acute myeloid leukemia survivors: A single-center study. Eur J Haematol. 2014;93(3).

13. Hevér N V., Péntek M, Balló A, Gulácsi L, Baji P, Brodszky V, *et al.* Health Related Quality of Life in Patients with Bladder Cancer: A Cross-Sectional Survey and Validation Study of the Hungarian Version of the Bladder Cancer Index. Pathol Oncol Res. 2015;21(3).

14. Endarti D, Riewpaiboon A, Thavorncharoensap M, Praditsitthikorn N, Hutubessy R, Kristina SA. Evaluation of health-related quality of life among patients with cervical cancer in Indonesia. Asian Pacific J Cancer Prev. 2015;16(8).

15. Elaboração própria baseada em entrevistas com profissionais de saúde.

S6. Análise de sensibilidade para um intervalo de confiança de 95% para a elasticidade-preço e impacto acumulado do aumento de 50% de preços através de impostos em desfechos de saúde, nos custos e na arrecadação tributária em 10 anos.

**Elasticidade**	**- 0,4**	**- 0,48**	**- 0,6**
Mortes evitadas	120,897	145,077	181,346
Doenças cardíacas evitadas	94,514	113,417	141,771
AVC evitados	81,118	97,342	121,677
Novos casos de câncer evitados	55,296	66,355	82,943
Novos casos de DPOC evitados	225,607	270,729	338,411
Anos de vida perdidos por morte prematura e por incapacidade evitados	4,593,399	5,512,078	6,890,098
Custo direto da assistência médica evitado (R$ milhões)	32,001	64,002	96,003
Custo por produtividade perdida de cuidadores informais evitado (R$ milhões)	17,502	35,003	52,505
Perda evitada de produtividade (R$ milhões)	24,102	4,818	72,234
Aumento na arrecadação tributária (R$ milhões)	17,318	26,009	26,072
Benefício econômico total (R$ milhões)	90,923	173,195	246,814

AVC: acidente vascular cerebral; DPOC: doença pulmonar obstrutiva crônica.
